# Validation of a Frailty Ladder Using Rasch Analysis: If the Shoe Fits

**DOI:** 10.1093/geroni/igab046.1480

**Published:** 2021-12-17

**Authors:** Nancy Mayo, Mylène Aubertin-Leheudre, Kedar Mate, Sabrina Figueiredo, Julio Fiore, Mohammad Auais, Susan Scott, José Morais

**Affiliations:** 1 McGill University, Montreal, Quebec, Canada; 2 Universite du Quebec à Montreal, Montreal, Quebec, Canada; 3 Queen's University, kingston, Ontario, Canada; 4 McGill University Health Centre Research Institute, Montreal, Quebec, Canada

## Abstract

The current measurement approach to frailty is to classify people on frailty status, rather than measure the degree to which they are frail. Here, we test the extent to which a set of items identified within the frailty concept fits a hierarchical linear model (Rasch model) and form a true measure reflective of the frailty construct and confirm the model using the NuAge dataset. The development sample included 234 individuals (aged 57 to 97) drawn from three sources: at-risk seniors (n=141); post-colorectal surgery (n=47); and post-rehabilitation hip fracture (n=46). We defined our frailty construct based on items commonly used in frailty indices, self-report measures, and performance tests. Of the 68 items, 29 fit the Rasch Model: 19 self-report items on physical function and 10 performance tests including one for cognition. Items typically identified as reflecting the frailty concept fit the Rasch model. The Frailty Ladder would facilitate personalized intervention.

